# Single-cell transcriptional landscape of peripheral myeloid cells in autoimmune diseases

**DOI:** 10.1016/j.isci.2025.113026

**Published:** 2025-07-01

**Authors:** Frank Qingyun Wang, Xiao Dang, Caicai Zhang, Huidong Su, Yao Lei, Xinxin Chen, Jing Yang, Wanling Yang

**Affiliations:** 1 Department of Paediatrics and Adolescent Medicine, The University of Hong Kong, Hong Kong, China

**Keywords:** Immunology, Components of the immune system, Transcriptomics

## Abstract

Myeloid cells are pivotal in autoimmune disorder development due to their varied immune response functions yet understanding them at the single-cell level in such conditions is limited. To address this gap, we analyzed 351,905 myeloid cells from 375 single-cell RNA sequencing samples covering 14 autoimmune diseases, which allowed us to classify myeloid cells into 13 distinct subsets with unique immunological profiles. Through pseudotime analysis, we identified a branching process leading to two intermediary inflammatory subtypes and observed dysregulation in eight co-expression modules crucial for monocyte function across autoimmune conditions. Notably, a module linked to myeloid cell activation was consistently upregulated in autoimmune disorders. This insight led us to identify potential drug targets for therapeutic repurposing within the dysregulated modules. By incorporating genetic data from genome-wide association studies, we identified 13 myeloid cell subsets predisposed to varying degrees of risk in different autoimmune conditions.

## Highlights

• Identified 13 functionally distinct myeloid subsets and 8 monocyte cellular states
• Revealed cellular states disrupted in autoimmunity and pinpointed drug targets
• Myeloid subsets significantly enriched heritability of autoimmune diseases

## Graphical abstract

**Figure:**
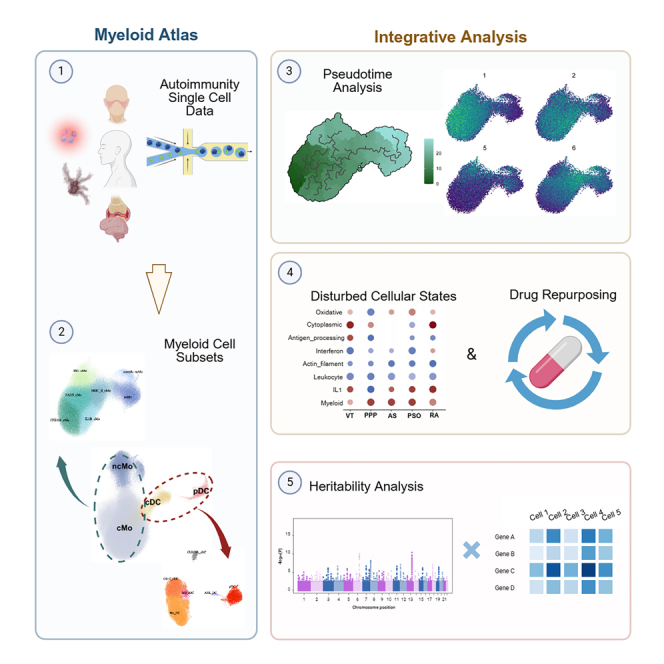

## Introduction

Autoimmune disorders manifest when the immune system erroneously targets and attacks the body’s own tissues, resulting in a spectrum of health complications. The pathogenesis of these conditions is intricate and multifaceted, involving genetic predispositions, environmental triggers, and potential infections.^1^ This misdirected immune response is often linked to a breakdown in immunologic tolerance, where autoreactive immune cells are inadequately suppressed.

Myeloid cells play a pivotal role in the pathogenesis of autoimmune disorders due to their dual functions in immune response regulation.^2^ Comprising monocytes, macrophages, and dendritic cells, these cells are essential for initiating immune responses and maintaining tissue homeostasis. In the context of autoimmunity, dysregulation of myeloid cells can lead to the excessive production of pro-inflammatory cytokines such as tumor necrosis factor alpha (TNF-α), interferon, and interleukin-6 (IL-6).^3^^,^^4^^,^^5^ This overproduction establishes a feedback loop that perpetuates chronic inflammation, resulting in the characteristic symptoms of autoimmune diseases, including pain, swelling, and tissue damage.^6^ Additionally, myeloid cells are crucial for phagocytosis, a process that influences immune tolerance and inflammatory regulation. Under normal circumstances, they efficiently clear apoptotic cells and debris, promoting the release of anti-inflammatory cytokines like IL-10 and transforming growth factor beta (TGF-β), which help maintain tolerance to self-antigens.^7^ However, when phagocytic function is impaired, as often observed in autoimmune diseases, the accumulation of apoptotic cells can activate self-reactive lymphocytes, exacerbating inflammation.^8^^,^^9^

The multifaceted role of myeloid cells underscores the heterogeneity of this cell population. Understanding the subtypes of myeloid cells is crucial for comprehending their diverse roles in the immune system. Given the pivotal role of myeloid cells in autoimmunity pathogenesis, they represent promising targets for the development of novel therapies. Consequently, identifying dysregulated programs in myeloid cells and prioritizing potential drug targets is essential. However, a comprehensive evaluation of the myeloid cell landscape in autoimmunity is currently lacking, impeding a thorough understanding of their functionalities.

Recent studies have increasingly leveraged single-cell RNA sequencing (scRNA-seq) technology to explore the immune landscape in patients with autoimmunity. This technology offers insights into the distinct transcriptional profiles of individual cells, enabling the detailed characterization of cell heterogeneity. Through this approach, dysregulated immune pathways and associated cell types in disease conditions have been elucidated. Moreover, advancements in analytic techniques allow for the integration of cells from large-scale studies that are readily accessible.^10^ In this study, we conducted a comprehensive analysis of 351,905 peripheral myeloid cells from 375 donors across 14 autoimmune conditions. Our findings demonstrate the classification of peripheral myeloid cells into subsets with distinct functions. The differential dysregulation of monocyte cellular states in each autoimmunity condition highlights potential therapeutic targets. Furthermore, by integrating summary statistics from genome-wide association studies (GWASs) with our integrated myeloid cell dataset, we identified genetically predisposed etiological cell subsets. These analyses would facilitate our understanding of myeloid cells in autoimmunity conditions.

## Results

### Integration of myeloid-derived immune cells across autoimmune diseases

**Figure 1:**
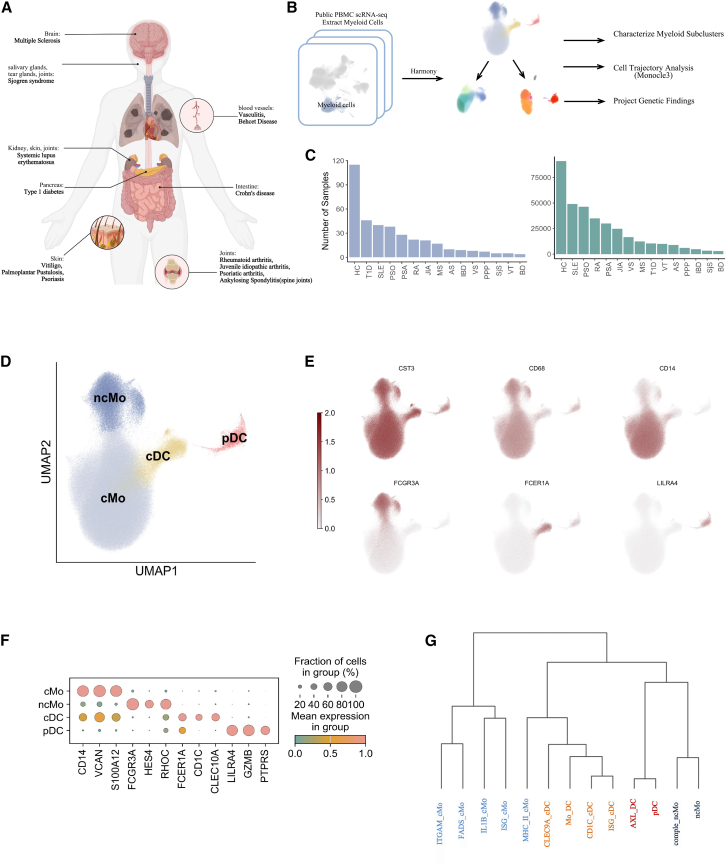
Integration of peripheral myeloid cells from autoimmunity conditions (A) Autoimmunity conditions included in this study. (B) Workflow of the analytic strategies. (C) Number of samples (left) and cells (right) included in this study. (D) UMAP plot showing the annotation of the myeloid cells based on classical immunological markers. (E) UMAP plots showing the expression of major marker genes. (F) Dotplot showing the expression of markers gene in each cell type. (G) The relationship between the cell subsets shown by the dendrogram.

To have a thorough repertoire of peripheral myeloid-derived immune cells in autoimmune diseases, we obtained scRNA-seq data from 375 individuals, comprising 115 healthy controls and 260 patients diagnosed with one of the 14 common autoimmune conditions: systemic lupus erythematosus (SLE), multiple sclerosis (MS), Behçet disease, psoriatic arthritis, psoriasis (PSO), ankylosing spondylitis (AS), Crohn disease, vasculitis, vitiligo, type 1 diabetes, juvenile idiopathic arthritis (JIA), palmoplantar pustulosis, rheumatoid arthritis (RA), and Sjögren syndrome (SjS) (Figures 1A–1C). We utilized the gene markers CD68 and CST3, known for their universal expression in myeloid cells, to isolate the cell clusters of myeloid cell origin within each dataset (Figure 1B). Stringent quality control was applied to remove low-quality cells and doublets, and batch effect was corrected across the datasets using Harmony,^10^ which resulted in 351,905 cells for the subsequent analysis (see Figures S1 and S2 and Table S5).

The initial round of dimension reduction and unsupervised clustering reveals four primary cell types based on canonical gene markers (Figure 1D) (see Table S2). The major cell types identified are classical monocytes (*n* = 268,976), characterized by CD14^+^, and non-classical monocytes (*n* = 50,099), distinguished by FCGR3A+. These two groups constitute the largest segments within the peripheral myeloid populations. The remaining cell types include classical dendritic cells (*n* = 26,048), recognized by CD1C+, and plasmacytoid dendritic cells (pDCs) (*n* = 6,782), identified by LILRA4+ (Figures 1E and 1F). In the subsequent analysis, an additional 13 myeloid cell subtypes were identified. The dendrogram highlights a closer relationship between the various monocyte cell types, with the exception of MHC_II_monocytes and nonclassical monocytes, which show greater proximity to the dendritic cells.

### Myeloid-derived cells population are heterogeneous and exhibits distinct function

**Figure 2:**
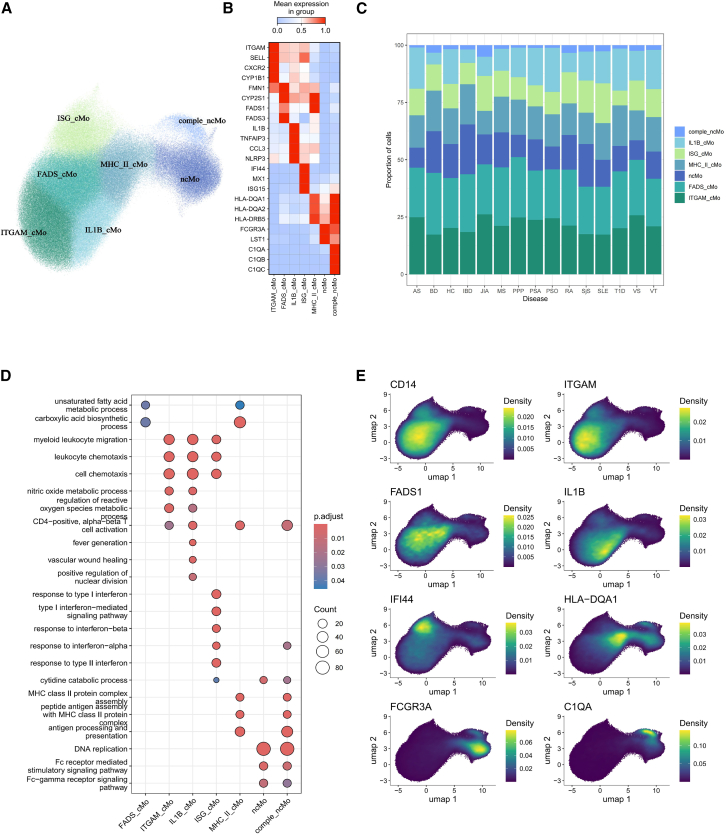
Characterization of monocytes subsets (A) UMAP plot showing the monocytes subsets. (B) Heatmap showing the scaled gene signature for each subset. (C) Bar plot showing the proportions of monocytes across different conditions. (D) Dotplot showing the functional enrichment analysis result of each subset using gene ontology biological process database. (E) Visualization of the expression density of marker genes on UMAP.

We have identified seven subpopulations of monocytes, each representing a distinct functional state (Figure 2A). The ITGAM_cMo subgroup (*n* = 69,084) is characterized by high expression of S100 family genes, CXCR2, and SELL (Figure 2B), which play roles in the activation, taxis, and regulation of the oxygen species process in monocytes. The FADS_cMo group (*n* = 71,185) is the largest group of monocytes exhibiting high expression of the fatty acid desaturase (FADS) family gene, which is involved in fatty acid metabolism and the carboxylic acid biosynthetic process. Functional enrichment analysis also indicates immune-related pathways are not enriched in this cell type, suggesting a less inflammatory state (Figure 2D). Conversely, two distinct highly inflammatory monocyte groups have been identified. The first, named ISG_cMo (*n* = 38,544), is characterized by hyperactivated interferon gene expression, such as IFI44, MX1, and ISG15, and is predominantly enriched in the type 1 interferon pathways (Figure 2D). The second group, IL1B_cMo (*n* = 45,159), is marked by high levels of proinflammatory cytokines including IL1B, NLRP3, and CCL3. Additionally, we identified a group of MHC_II_cMo (*n* = 46,825) marked by high expression of MHC class II molecules, such as HLA-DQA1 and HLA-DRB5, which resembles the characteristics of well-known intermediate monocytes.^11^ Furthermore, among non-classical monocytes characterized by high expression of FCGR3A and upregulated Fc-receptor-mediated stimulatory signaling pathways, we identified a small subgroup of these comple_ncMo (*n* = 6,949) distinguished by complement molecules such as C1QA, C1QB, and C1QC (Figures 2D and 2E) (see Figure S3A).

**Figure 3:**
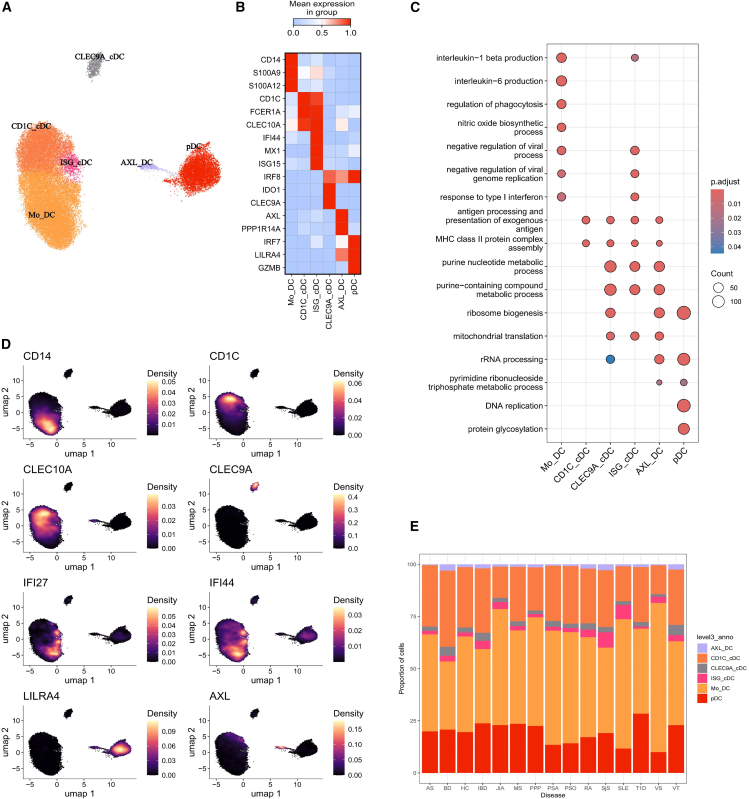
Characterization of dendritic subsets (A) UMAP plot showing the dendritic cell subsets. (B) Heatmap showing the scaled gene markers for each subset. (C) Dotplot showing the functional enrichment analysis result of each subset using gene ontology biological process database. (D) Density plot visualization of the expression of marker genes. (E) Visualization of the expression density of marker genes on UMAP.

We classified the dendritic cells into six groups (Figure 3A). The Mo_DC group (*n* = 16,477) emerges as the largest, characterized by the expression of CD14 and S100 family genes, suggesting a monocyte origin. Functional enrichment analysis indicates that genes upregulated in Mo_DC are enriched in pathways associated with IL-1β production, IL-6 production, and chemotaxis, indicating a more inflammatory state than the other dendritic cells. The CD1C_cDC group (*n* = 8,350) is known for expressing markers typical of conventional type 2 dendritic cells, such as CD1C, FCER1A, and CLEC10A. The pDC group (*n* = 5,864) is distinguished by canonical markers like IRF7, LILRA4, and GZMB (Figures 3B and 3D). Genes upregulated in pDCs are enriched in cell-cycle pathways such as DNA replication (Figure 3C). Noteworthy, our integration strategy and meta-analysis have enabled the identification of cell subtypes that were previously indistinguishable in individual studies due to their limited representation in each sample (Figure 3E). We identified a CLEC9A_cDC group (*n* = 785) characterized by the expression of markers associated with conventional type 1 dendritic cells, including IDO1 and CLEC9A. The ISG_cDC group (*n* = 938) is characterized not only by the expression of CD1C and FCER1A but also by the high expression of interferon genes including IFI27 and IFI44. Samples from SjS and SLE show the highest proportions of ISG_cDC, highlighting the important role of type 1 interferon pathway in these autoimmunity conditions (Figure 3E). Beyond, the smallest group of dendritic cells, AXL_DC (*n* = 416), which highly express AXL and PPP1R14A, is identified (Figures 3B and 3D). All CLEC9A_cDC, ISG_cDC, and AXL_DC exhibit functional similarities enriched in the purine nucleotide metabolic process and mitochondrial translation. Additionally, CLEC9A_cDC, AXL_DC, and pDC are enriched in pathways related to functions of ribosomes (Figure 3C) (see Figure S3B).

**Figure 4:**
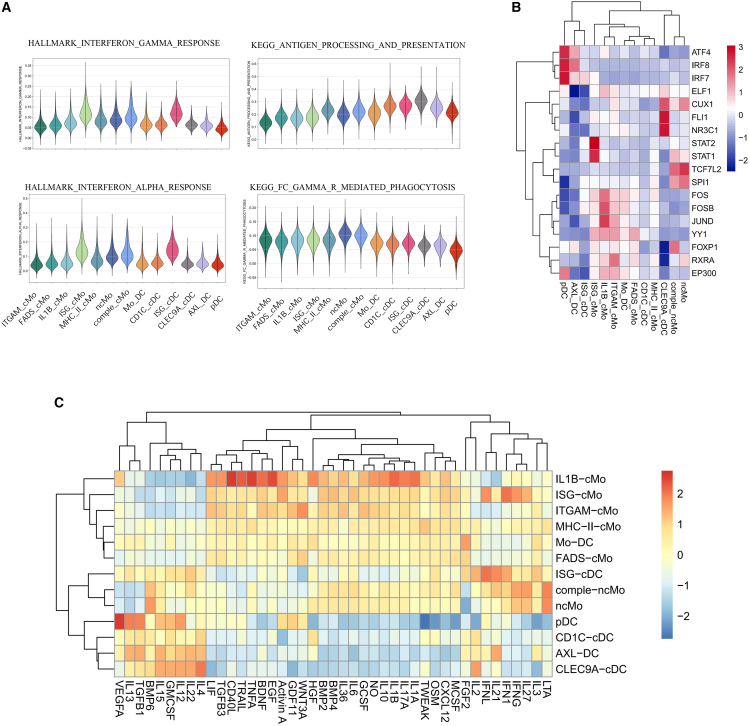
Myeloid cell subsets exhibit diverse phenotypes (A) Violin plot showing the expression level of selected functions across the myeloid cell subsets. (B) Heatmap showing the scaled transcription factor activity inferred from SCENIC for each cell subset (SCENIC: single-cell regulatory network inference and clustering). (C) Heatmap showing the scaled cytokine responsiveness inferred from CytoSig for each subset.

We further delineate the distinct functional roles of the 13 subtypes. ISG_cMo and ISG_cDC exhibit the highest interferon alpha and interferon gamma signatures (Figure 4A) and consistently display a robust response to IFN1, IFNL, and IFNG cytokines (Figure 4C). Leveraging the SCENIC workflow,^12^ we identified STAT1 and STAT2 as the key regulons that drive the program of ISG_cMo (Figure 4B). IL1B_cMo, on the other hand, demonstrates the highest responsiveness to a wide range of cytokines, including TNFA, IL6, IL1B, and CD40L, underscoring its inflammatory function (Figure 4C). The master regulons defining IL1B_cMo comprise FOS, FOSB, and JUND, highlighting their significance in the inflammatory response (Figure 4B). Both ncMo and comple_ncMo are characterized by elevated FC-gamma-receptor-mediated phagocytosis signatures, emphasizing their crucial role in antigen uptake and clearance of apoptotic cells (Figure 4A), with TCF7L2 and SPI1 identified as the master regulators for both cell types (Figure 4B).

Regarding the dendritic cells, Mo_DC shows a similar cytokine response pattern to other monocytes, indicating functional similarities (Figure 4C). In contrast, the remaining dendritic cell subtypes exhibit a stronger response to a distinct set of cytokines such as IL-4 and IL-13. Overall, dendritic cells exhibit a higher potential for antigen processing and presentation compared to monocytes, with CLEC9A_cDC and CD1C_cDC among the top (Figures 3C and 4A). SCENIC analysis reveals that pDC and AXL_DC are regulated by similar regulons including ATF4, IRF7, and IRF8, whereas CLEC9A_cDC is governed by CUX1, FL1, and NR3C1 (Figure 4B).

### Trajectory analysis reveals developmental path of monocytes

**Figure 5:**
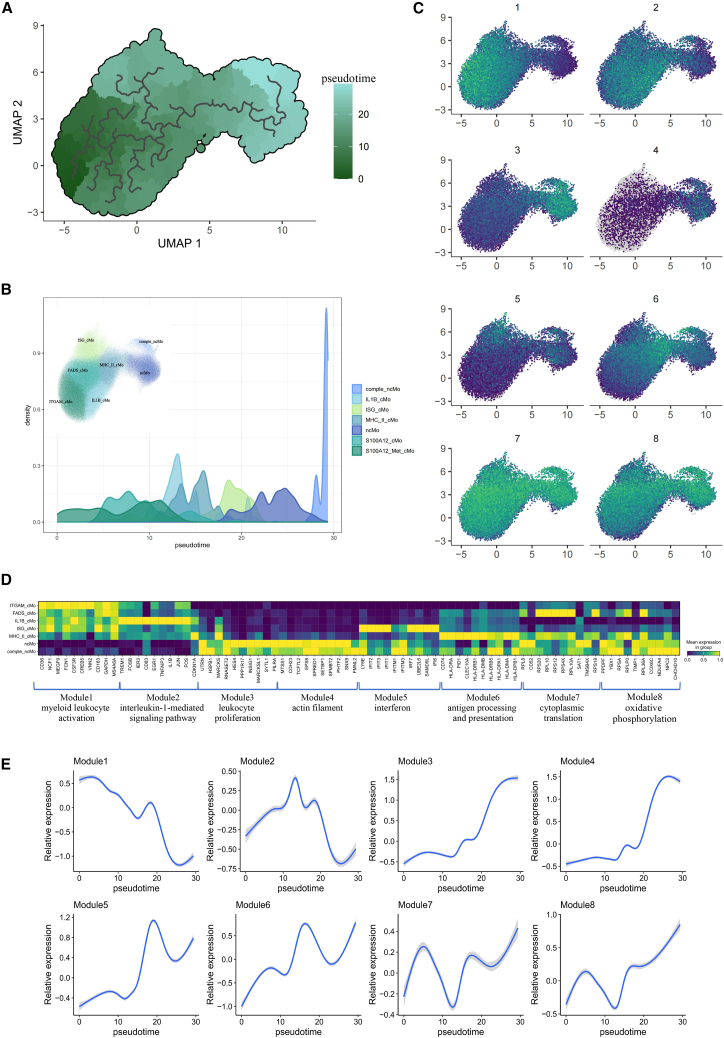
Pseudotime analysis of monocytes (A) UMAP color-coded by the pseudotime. (B) Density plot showing the pseudotime distribution across the previously described subsets. (C) UMAP plots showing the eight co-expression modules. (D) Heatmap showing the expression of top 10 genes in each module in each monocyte subset. (E) Expression trend of eight modules throughout the imputed pseudotime.

The transition from classical monocytes (CD14^+^) to non-classical monocytes (CD16^+^) is a well-documented process in immunology. To explore the intricate differentiation trajectory within this transition, we conducted pseudo-time analysis using Monocle3.^13^ We designated ITGAM_cMo as the “root” due to its minimal expression of markers (MAFB, RHOC, and CSF1R) involved in monocyte differentiation (see Figure S5). Along the developmental path from ITGAM_cMo toward becoming non-classical monocytes, MHC_II_cMo are positioned in the intermediate stage. Notably, IL1B_cMo and ISG_cMo emerge as two distinct branched states during this differentiation process, indicating a different developmental state of classical monocytes (Figures 5A and 5B).

Subsequently, we identified pseudotime-dependent genes and categorized them into eight co-expression modules highlighting the most essential functions underlying monocytes (Figure 5B) (see Figure S4A and Table S3). Genes in module 1, such as NCF1, CD36, SELL, CCR1, and CCR2, are enriched in chemotaxis and the regulation of reactive oxygen species metabolic processes, primarily expressed in the early developmental trajectory (Figures 5D and 5E). Modules 2, 5, and 6 exhibit the highest expression in the intermediate developmental trajectory (Figure 5E). Notably, IL1B_cMo shows the highest expression in module 2, which includes genes involved in the IL-1-mediated signaling pathway, such as IL-1β, CCL3, and TNFAIP3. Module 5 comprises interferon-related genes like IFI44, ISG15, and MX1, whereas module 6 consists of major histocompatibility complex class II (MHC-II)-associated genes such as HLA-DRA, HLA-DRB5, and HLA-DQA1 (Figure 5D). Conversely, non-classical monocytes highly express genes from modules 3 and 4 at high levels (Figures 5C and 5D). Module 3 includes genes like TNFRSF14, CD86, and RUNX3, predominantly enriched in myeloid cell differentiation and leukocyte proliferation. Module 4 encompasses genes such as ADD2, SNX9, and EPS8, which function in the actin filament. Genes in modules 7 and 8 are universally expressed across cells. Module 7 contains highly expressed genes like RPL20, RPL10, and RPL8, enriched in ribosomal functions and cytoplasmic translation, with the highest level observed in the FADS_cMo and MHC_cMo. Module 8, enriched in oxidative phosphorylation and mitochondrial electron transport, shows the highest expression in non-classical monocytes (Figure 5C).

### Eight cellular programs in monocytes are dysregulated across autoimmune diseases

**Figure 6:**
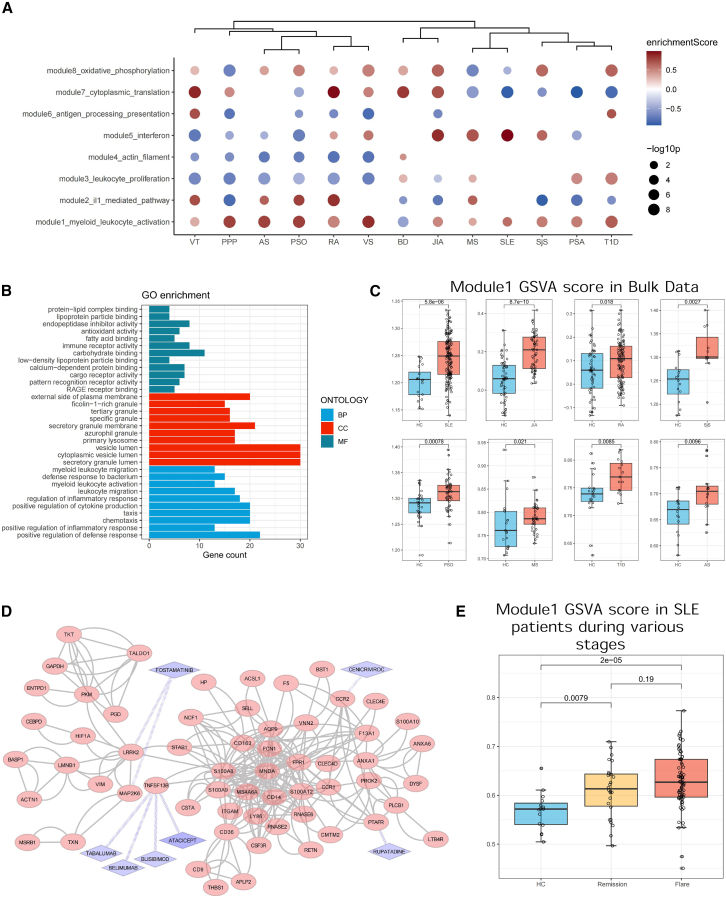
Dysregulation of eight monocyte co-expression modules across autoimmune diseases (A) Dotplot showing the GSEA results comparing the autoimmunity conditions to the healthy controls based on the eight co-expression modules. The color denotes the enrichment score, whereas the dot size denotes the -log10 *p* value (GSEA: gene set enrichment analysis). (B) Barplot showing the GO biological process terms enriched in the genes of module 1 (GO: gene ontology). (C) Boxplots showcasing the significantly upregulation (Wilcoxon rank-sum test) of module 1 GSVA score across autoimmunity conditions validated in the bulk transcriptomic data (GSVA: gene set variation analysis). (D) Protein-protein interaction network presenting the highly connected genes from module 1 and their potential drug targets retrieved from the ChEMBL and DrugBank database. (E) Boxplots display the differential expression of module 1 in healthy controls versus SLE patients (remission and flare stages), with significance assessed by Wilcoxon rank-sum test.

Our subsequent goal is to elucidate the functional changes of monocytes in various autoimmune conditions. We hypothesize that the functional state of monocytes undergoes continuous changes due to their developmental nature. Therefore, rather than simply comparing the cell number proportions of each cell subtype, we assessed the disruptions of the eight identified co-expression modules in each autoimmune condition using gene set enrichment analysis (Figure 6A).

It can be seen that eight co-expression modules are broadly dysregulated across the autoimmune diseases compared to the controls. The interferon-associated module 5 is elevated in RA, VS, JIA, MS, SLE, and SjS, whereas the IL-1-mediated module 2 is upregulated in ventricular tachycardia (VT), AS, PSO, RA, and MS. Modules 4 and 5, which are highly expressed in non-classical monocytes, are consistently downregulated in VT, PPP, AS, PSO, RA, and VS. This analysis highlights distinct immune pathways influencing varying monocyte activation and suggests diverse disturbances in monocyte functions across autoimmune diseases.

Remarkably, module 1 exhibits a consistent pattern of upregulation across a spectrum of autoimmune diseases, with the exception of Behcet disease (Figure 6A). Upon conducting gene ontology enrichment analysis, we discovered that module 1 is particularly enriched with functions related to chemotaxis, regulation of defense response, and granule-related activities (Figure 6B). To further validate the expression pattern of module 1, we employed gene set variation analysis to quantify its levels in PBMC samples obtained from bulk transcriptomic data spanning different autoimmune diseases. Our analysis revealed a significant increase in the expression of module 1 in patients with SLE, JIA, RA, SjS, PSO, MS, T1D, and AS compared to healthy controls, consistent with the observations from single-cell data. Moreover, we used an in-house SLE data to show that the SLE patients exhibit significantly elevated expression levels of module 1 compared to healthy controls (Wilcoxon rank-sum test). Interestingly, patients in remission and flare stages both displayed similar expression levels of module 1, emphasizing its consistent dysregulation in various stages of autoimmunity (Figure 6E). This observation underscores the universal upregulation of module 1 as a shared feature across diverse autoimmune conditions, suggesting its role in the immune hyperactivation in autoimmunity (Figure 6C).

The disturbed module expression, especially consistent activation of module 1 and in various autoimmune diseases, highlights the genes within this module as promising therapeutic candidates (see Figure S6). A protein-protein interaction network is created using the STRING database to illustrate the interconnected functionality of genes in module 1 (Figure 6D). Subsequently, we utilize the ChEMBL and DrugBank database to identify phase III-/IV-approved bioactive molecules targeting the genes in the network, which are used to treat at least one type of autoimmune diseases. TNFSF13B is targeted by four drugs (Tabalumab, Belimumab, Atacicept, and Blisibimod), also known as B cell activation factor (BAFF), which promotes B cell proliferation and survival. It is suggested that these drugs can be repurposed to treat a broader spectrum of autoimmune diseases according to its pervasive role in autoimmunity.

### Myeloid cells enrich heritability of complex traits

**Figure 7:**
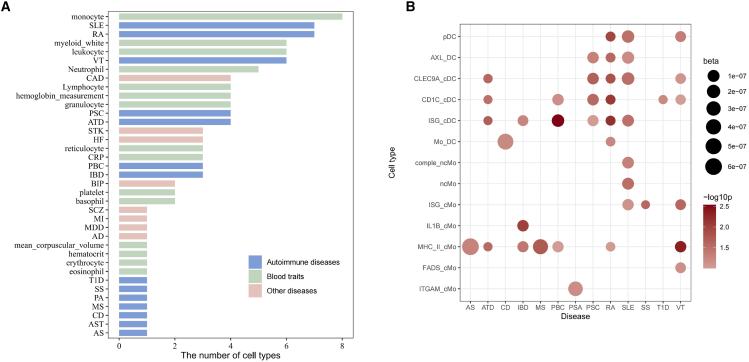
Heritability analysis of complex traits in myeloid cell subsets (A) Barplot illustrating the number of myeloid cell subsets significantly enriched with each feature (SLE, systemic lupus erythematosus; RA, rheumatoid arthritis; VT, vitiligo; CAD, coronary artery disease; PSC, primary sclerosing cholangitis; ATD, autoimmune thyroid disease; STK, stroke; HF, heart failure; CRP, C-reactive protein; PBC, primary biliary cholangitis; IBD, inflammatory bowel disease; BIP, bipolar disorder; SCZ, schizophrenia; MI, myocardial infarction; MDD, major depressive disorder; AD, Alzheimer disease; T1D, type 1 diabetes; SS, systemic sclerosis; PA, psoriatic arthritis; MS, multiple sclerosis; CD, celiac disease; AST, asthma; AS, ankylosing spondylitis). (B) Dotplot displaying the heritability of autoimmune diseases enriched in each myeloid cell subset. Dot size represents the beta value while dot color represents the -log10 *p* value derived from the S-LDSC.

We then investigate the correlation between the 13 identified cell subtypes and the genetic factors associated with complex traits identified in GWAS studies. We used the top 10% genes highly expressed in each cell type as representative cell-type-specific feature genes. By employing the CELLECT pipeline, which utilizes S-LDSC to prioritize the etiologic cell types for a given human complex trait, we demonstrate that the heritability of complex traits is enriched with myeloid cell subtypes (see Table S4). The heritability of monocyte count exhibits the highest enrichment among myeloid cell subsets, validating the alignment between genetic discoveries and transcriptomic characteristics, with other cell count features following along such as leukocyte count, myeloid white cell count, and neutrophil count (Figure 7A).

Heritability of several autoimmunity diseases is enriched in the myeloid cell programs, suggesting the pathogenesis of autoimmunity is closely related with the myeloid cells (Figure 7B). Overall, our analysis revealed that the heritability of autoimmune diseases is more enriched in the MHC_II_cMo and dendritic cell subsets including CD1C_cDC, CLEC9A_cDC, and ISG_cDC, compared to other monocyte subtypes. This is primarily attributed to the fact that dendritic cells and MHC_II_cMo exhibit the highest expression levels of MHC class II genes, with MHC class II genes in the HLA regions showing the most significant signals in GWAS studies of autoimmune diseases. Specifically, SLE stands out as the disease with the most association with a wide range of cell types, highlighting the crucial role of myeloid cells in SLE susceptibility. In terms of monocytes, apart from the broad susceptibility observed in MHC_II_cMo, IL1B_cMo predispose the risk of inflammatory bowel disease, ITGAM_cMo predispose the risk of psoriatic arthritis, and FADS_cMo predispose the risk of vitiligo. Furthermore, ISG_cMo subtype predispose risk of SLE, SS, and VT, showcasing the importance of interferon-related pathway in these diseases.

## Discussion

Myeloid cells are pivotal in the pathogenesis of various autoimmune conditions, yet our comprehension of their functionality and diversity remains incomplete. Integrative analysis of single-cell data from multiple sources has shed light on the origins of immune cells and their association with various disease states.^14^^,^^15^ In this study, we integrated expression profiles of peripheral myeloid cells across diverse autoimmune conditions to illuminate the multifaceted roles of these cells and pinpoint dysregulated cellular programs of each autoimmune disorder. Our comprehensive analysis encompasses over 350,000 myeloid cells from 14 autoimmunity conditions. Through a combination of supervised and unsupervised approach, we classified the myeloid cells into 13 subtypes with distinct functions.

Monocytes are essential players in immune responses, and their differentiation trajectory—from classical (CD14^+^) to non-classical monocytes (CD16^+^)—reflects their evolving roles in maintaining immune homeostasis. This transition is more than a mere change in cell surface markers; it mirrors profound changes in monocyte function. The use of pseudotime analysis in this study revealed not just the progression from classical to non-classical monocytes but also a branching process where intermediate and specialized subpopulations of classical monocytes, such as IL1B_cMo and ISG_cMo, emerge. Identification of eight co-expression modules corresponding to the pseudotime sheds light on the complex gene networks that regulate monocyte function. These modules not only represent different stages of monocyte development but also reflect distinct functional programs that are dysregulated in the context of autoimmune diseases.

Module 1, which is consistently upregulated across a wide range of autoimmune diseases, is particularly interesting. The persistent activation of this module in autoimmune diseases suggests that these monocytes may be “stuck” in a hyperactivated state, perpetuating inflammation. This module is enriched in genes involved in chemotaxis (CCR2, SELL, CCR1), myeloid cell activation, and reactive oxygen species regulation (ITGAM, CLU, CD36), which are crucial in mounting an effective immune response. The activated peripheral monocytes in autoimmunity are more prone to be recruited to inflamed tissues in response to injury-associated signals, including pro-inflammatory cytokines.^8^^,^^16^^,^^17^^,^^18^ This recruitment leads to their differentiation into macrophages, which are essential for initiating and propagating tissue-specific immune responses. The persistent activation of module 1 across multiple autoimmune diseases points to a common molecular pathway that could be targeted to alleviate inflammation in a wide range of conditions. The PPI network constructed around genes in this module highlights TNFSF13B, a cytokine involved in B cell survival and proliferation, as a key player. Given that BAFF-targeting drugs (e.g., Belimumab and Atacicept) are already approved for the treatment of autoimmune diseases like SLE, this finding suggests that these therapies could potentially be repurposed or expanded to treat other conditions where module 1 is upregulated.^19^

Similarly, module 2 and 5 also play distinct roles in autoimmune diseases. Module 2, enriched in IL-1 signaling, aligns with the pro-inflammatory IL1B_cMo monocyte state. This module is upregulated in diseases like vasculitis, AS, and PSO, indicating that IL-1-driven inflammation is a common pathological feature in these diseases. The elevation of interferon-associated module 5 in diseases such as RA, MS, and SLE further emphasizes the role of type I interferons in driving monocyte activation in autoimmunity. This heterogeneity in monocyte function has important implications for treatment. It suggests that therapies targeting monocytes need to be tailored to the specific disease context, considering which functional programs are dysregulated.^20^

Our integrative analysis with large sample sizes allows identification of cell types with limited representations in each individual. ISG_cDC exhibits hyperactivation of interferon-stimulated genes (ISGs). This subset shares some functional characteristics with the ISG_cMo monocyte group, particularly its heightened response to interferon signaling. Notably, ISG_cDCs were found in higher proportions in samples from patients with SjS and SLE, two diseases where type I interferon pathways are known to be central.^21^^,^^22^

Moreover, other dendritic cell subsets identified, such as CLEC9A_cDC and AXL_DC, display less inflammatory features compared to Mo_DC and ISG_cDC. CLEC9A_cDC, characterized by markers of conventional type 1 DCs, is enriched in antigen processing and presentation pathways. Under normal conditions, cDC1 can contribute to immune tolerance by presenting self-antigens in a non-inflammatory context, promoting the deletion or anergy of autoreactive T cells.^23^^,^^24^ The ability of cDC1 to engage in cross-presentation also makes them pivotal in the recognition of dead or dying cells.^24^ However, when tolerance mechanisms fail or are overwhelmed, especially in autoimmunity, cDC1 may inadvertently support the activation of these autoreactive cells, fueling autoimmune pathology.^25^

AXL dendritic cells, the smallest group of dendritic cells identified in our study, are a recently identified subset of dendritic cells with unique properties, and their role in autoimmunity is an emerging area of research. AXL_DC is characterized by the expression of AXL, a receptor tyrosine kinase involved in various immune regulatory pathways, particularly those inhibiting excessive immune responses and promoting tolerance by preventing overactivation of the immune system.^26^ They are also known for promoting phagocytosis of apoptotic cells and secreting anti-inflammatory cytokines.^27^ In autoimmune diseases, their function could be compromised, leading to impaired regulation, insufficient clearance of apoptotic cells, and dysregulated interferon responses, contributing to disease progression. However, limited research was conducted to elucidate the functional role of those underrepresented cells in autoimmunity.

It is important to note that within this myeloid cell atlas, approximately 20% of pDCs potentially originate from a lymphoid lineage.^28^^,^^29^ In our analysis, we were unable to differentiate between pDCs derived from lymphoid progenitors and thus categorized all identified dendritic cells as myeloid cells. Future research could explore the specific investigation of lymphoid-derived pDCs in scRNA-seq analyses to further elucidate their distinct characteristics and origins.

The genetic architecture of autoimmune diseases is complex and multifactorial, involving numerous loci that influence immune system behavior. Combining with the genetic findings, we are able to prioritize the myeloid subtypes that predispose the risk of complex traits. Variants within the MHC region, particularly those affecting MHC class II molecules, are strongly associated with susceptibility to autoimmunity.^30^^,^^31^ MHC class II molecules are crucial for presenting processed peptides, including self-antigens, to CD4^+^ T cells. In the context of autoimmunity, aberrations in this process can lead to the activation of autoreactive T cells. Given the high expression of MHC genes in MHC_II monocytes and dendritic cells, they are significantly enriched in wide range of various autoimmunity conditions, emphasizing their importance in initiating and shaping the susceptibility of autoimmunity. Moreover, we identified interferon-producing classical monocytes significantly enriched heritability of SLE, systemic sclerosis, and vitiligo, aligning with the previous findings that interferon plays pivotal role in these diseases.^32^ IL-1β classical monocytes significantly enrich the heritability of inflammatory bowel disease. It is known that elevated levels of IL-1β are frequently observed in patients with active IBD, correlating with disease severity and localized inflammation in the colon.^33^ Interestingly, while ITGAM classical monocytes showed the highest expression of module 1 genes, which were consistently upregulated across various autoimmune conditions, this cell type exhibited limited enrichment of heritability for complex traits. Instead of viewing this as a discordance, we interpret it as an intriguing phenomenon. GWAS analysis usually reveals susceptibility to a disease before its onset, suggesting that the identified susceptible cells may have a role prior to disease development. On the other hand, transcriptomic analysis centers on molecular features present in the patient’s current state, with the dysregulated module one being a notable finding during disease onset. These analyses offer distinct perspectives on the contribution of different cellular states to the pathogenesis of the disease in various stages.

In conclusion, we have delineated the landscape of peripheral myeloid cells in autoimmune diseases, serving as a foundational reference for future research aiming to comprehend the heterogeneity, functionality, and developmental pathways of these cells relevant to the autoimmune disease states. The identification of shared and unique dysregulated monocyte programs offers valuable biological insights for the potential development of novel drug targets focusing on these cells in the context of these autoimmunity. Moreover, the enrichment of heritability in complex traits suggests that various myeloid cell subsets play a role in the predisposing risk to these diseases.

### Limitations of the study

This study has several limitations. First, a lack of treatment information for each patient hinders our understanding of how the states of myeloid cells are linked to current therapeutic approaches in autoimmune disease. In addition, although pseudotime analysis provides valuable insights into monocyte cellular states and transitions, it is crucial to validate these *in silico* findings through experimental perturbation studies. We hope that future studies can further build upon this research to validate the cellular states and dysregulated modules in autoimmune conditions using an experimental approach.

## Resource availability

### Lead contact

Further information and requests for resources and data should be directed to and will be fulfilled by the lead contact, Wanling Yang (yangwl@hku.hk).

### Materials availability

This study did not generate any new unique reagents.

### Data and code availability

• This study did not generate any new data. All data are curated from existing public database with following accession. The scRNA-seq data^34^^,^^35^^,^^36^^,^^37^^,^^38^^,^^39^^,^^40^^,^^41^^,^^42^^,^^43^^,^^44^^,^^45^^,^^46^ are collected from Gene Expression Omnibus (GEO): systemic lupus erythematosus (SLE) (GSE13577), primary Sjögren’s syndrome (SjS) (GSE157278), multiple sclerosis (MS) (GSE138266, GSE133028), Crohn disease (CD) (GSE134809), Behcet disease (BD) (GSE198616), vitiligo (VT) (GSE231794), ankylosing spondylitis (AS) (GSE194315), psoriasis (PSO) (GSE194315), psoriatic arthritis (PSA) (GSE194315), palmoplantar pustulosis (PPP) (GSE185857), and juvenile idiopathic arthritis (JIA) (GSE207633). Additionally, data are collected from the Genomic Expression Archive (GEA) for vasculitis (E-GEAD-635) and from the Synapse database for type 1 diabetes (syn53641849). Raw sequencing data from the Genome Sequence Archive were obtained for rheumatoid arthritis (HRA000155) and subsequently processed by cell ranger to obtain the count matrix. The bulk array and RNA-seq data^47^^,^^48^^,^^49^^,^^50^^,^^51^^,^^52^^,^^53^^,^^54^ are collected from ankylosing spondylitis (GSE25101), multiple sclerosis (GSE203241), psoriasis (GSE55201), rheumatoid arthritis (GSE17755), juvenile idiopathic arthritis (GSE17755), Sjogren syndrome (GSE48378), systemic lupus erythematosus (GSE49454^53^), and type 1 diabetes (GSE72377).
• The established myeloid cells annotation model has been deposited in https://github.com/FrankQYW/Myeloid_Autoimmune_Ref. All code of the software is publicly available from R and python packages.
• Any additional information required to reanalyze the data reported in this work paper is available from the lead contact upon request.

## Acknowledgments

F.Q.W. thanks support from HKU Presidential PhD Scholar Program.

## Author contributions

Conceptualization, F.W. and W.Y. Data collection, F.W., C.Z., and X.C. Methodology, F.W., H.S., and X.D. Writing—original draft, F.W. and X.D. Writing—review & editing, W.Y., F.W., and Y.L. Supervision, J.Y. and W.Y. Funding acquisition, W.Y.

## Declaration of interests

The authors declare no competing interests.

## STAR★Methods

### Key resources table

**Table:** 

REAGENT or RESOURCE	SOURCE	IDENTIFIER
**Deposited data**		

Systemic lupus erythematosus (public single cell)	Nehar-Belaid et al.^34^	GEO:GSE135779 (https://www.ncbi.nlm.nih.gov/geo/query/acc.cgi?acc=GSE135779)
Primary Sjögren’s syndrome (public single cell)	Hong et al.^35^	GEO:GSE157278 (https://www.ncbi.nlm.nih.gov/geo/query/acc.cgi?acc=GSE157278)
Multiple sclerosis (public single cell)	Schafflick et al.^36^	GEO:GSE138266 (https://www.ncbi.nlm.nih.gov/geo/query/acc.cgi?acc=GSE138266)
Multiple sclerosis (public single cell)	Ramesh et al.^37^	GEO:GSE133028 (https://www.ncbi.nlm.nih.gov/geo/query/acc.cgi?acc=GSE133028)
Crohn’s disease (public single cell)	Martin et al.^38^	GEO:GSE134809 (https://www.ncbi.nlm.nih.gov/geo/query/acc.cgi?acc=GSE134809)
Behcet’s disease (public single cell)	Zheng et al.^39^	GEO:GSE198616 (https://www.ncbi.nlm.nih.gov/geo/query/acc.cgi?acc=GSE198616)
Vitiligo (public single cell)	Yang et al.^40^	GEO:GSE231794 (https://www.ncbi.nlm.nih.gov/geo/query/acc.cgi?acc=GSE231794)
Ankylosing Spondylitis (public single cell)	Liu et al.^41^	GEO:GSE194315 (https://www.ncbi.nlm.nih.gov/geo/query/acc.cgi?acc=GSE194315)
Psoriasis (public single cell)	Liu et al.^41^	GEO:GSE194315 (https://www.ncbi.nlm.nih.gov/geo/query/acc.cgi?acc=GSE194315)
Psoriatic arthritis (public single cell)	Liu et al.^41^	GEO:GSE194315 (https://www.ncbi.nlm.nih.gov/geo/query/acc.cgi?acc=GSE194315)
Palmoplantar pustulosis (public single cell)	McCluskey et al.^42^	GEO:GSE185857 (https://www.ncbi.nlm.nih.gov/geo/query/acc.cgi?acc=GSE185857)
Juvenile idiopathic arthritis (public single cell)	Verweyen et al.^43^	GEO:GSE207633 (https://www.ncbi.nlm.nih.gov/geo/query/acc.cgi?acc=GSE207633)
Type 1 diabetes (public single cell)	Honardoost et al.^46^	Synapse:syn53641849 (https://www.synapse.org/Synapse:syn53641849)
Rheumatoid arthritis (public single cell)	Wu et al.^44^	GSA:HRA000155 (https://ngdc.cncb.ac.cn/gsa-human/browse/HRA000155)
Vasculitis (public single cell)	Nishide et al.^45^	NBDC:E-GEAD-635 (https://ddbj.nig.ac.jp/public/ddbj_database/gea/experiment/E-GEAD-000/E-GEAD-635/)
Ankylosing Spondylitis (public array)	Pimentel-Santos et al.^47^	GEO:GSE25101 (https://www.ncbi.nlm.nih.gov/geo/query/acc.cgi?acc=GSE25101)
Multiple sclerosis (public array)	Shavit et al.^48^	GEO:GSE203241 (https://www.ncbi.nlm.nih.gov/geo/query/acc.cgi?acc=GSE203241)
Psoriasis (public array)	Wang et al.^49^	GEO:GSE55201 (https://www.ncbi.nlm.nih.gov/geo/query/acc.cgi?acc=GSE55201)
Rheumatoid arthritis (public array)	Lee et al.^50^	GEO:GSE17755 (https://www.ncbi.nlm.nih.gov/geo/query/acc.cgi?acc=GSE17755)
Juvenile idiopathic arthritis (public array)	Lee et al.^50^	GEO:GSE17755 (https://www.ncbi.nlm.nih.gov/geo/query/acc.cgi?acc=GSE17755)
Sjogren Syndrome (public array)	Sjostrand et al.^52^	GEO:GSE48378 (https://www.ncbi.nlm.nih.gov/geo/query/acc.cgi?acc=GSE48378)
Systemic lupus erythematosus (public array)	Chiche et al.^51^	GEO:GSE49454 (https://www.ncbi.nlm.nih.gov/geo/query/acc.cgi?acc=GSE49454)
Systemic lupus erythematosus (In house RNA-seq)	Frank et al.^53^	https://arthritis-research.biomedcentral.com/articles/10.1186/s13075-024-03327-4
Type 1 diabetes (public array)	Yamamoto et al.^54^	GEO:GSE72377 (https://www.ncbi.nlm.nih.gov/geo/query/acc.cgi?acc=GSE72377)

**Software and algorithms**		

Scanpy	Wolf et al. 2018^55^	https://github.com/scverse/scanpy
GEOquery	David et al. 2007^56^	https://github.com/seandavi/GEOquery
Limma	Ritchie et al. 2015^57^	https://github.com/cran/limma
Monocle3	Trapnell et al. 2014^13^	https://github.com/cole-trapnell-lab/monocle3
clusterProfiler	Wu et al. 2021^58^	https://github.com/YuLab-SMU/clusterProfiler
Nebulosa	Alquicira-Hernandez et al. 2021^59^	https://github.com/powellgenomicslab/Nebulosa
Symphony	Kang et al. 2021^60^	https://github.com/immunogenomics/symphony
GSVA	Hanzelmann et al. 2013^61^	https://github.com/rcastelo/GSVA
SCENIC	Aibar et al. 2017^12^	https://github.com/aertslab/SCENIC
CytoSig	Jiang et al. 2021^62^	https://github.com/data2intelligence/CytoSig
STRING	Szklarczyk et al. 2021^63^	https://string-db.org/
CELLECT	Timshel et al. 2020^64^	https://github.com/perslab/CELLECT
Decoupler	Badia et al.^65^	https://github.com/saezlab/decoupler-py
Cyoscape	Shannon et al.^66^	https://cytoscape.org/

### Method details

#### Data download and pre-processing

Pre-processed single-cell RNA-seq data from various conditions were sourced from different databases^34^^,^^35^^,^^36^^,^^37^^,^^38^^,^^39^^,^^40^^,^^41^^,^^42^^,^^43^^,^^44^^,^^45^^,^^46^ (see Table S1). The data from Gene Expression Omnibus (GEO) include: Systemic lupus erythematosus (SLE) (GSE13577), Primary Sjögren’s syndrome (SjS) (GSE157278), Multiple sclerosis (MS) (GSE138266, GSE133028), Crohn’s disease (CD) (GSE134809), Behcet’s disease (BD) (GSE198616), Vitiligo (VT) (GSE231794), Ankylosing Spondylitis (AS) (GSE194315), Psoriasis (PSO) (GSE194315), Psoriatic arthritis (PSA) (GSE194315), Palmoplantar pustulosis (PPP) (GSE185857), Juvenile idiopathic arthritis (JIA) (GSE207633). Additionally, data from the Genomic Expression Archive (GEA) for vasculitis (E-GEAD-635), and from the Synapse database for Type 1 diabetes (syn53641849). Raw sequencing data from the Genome Sequence Archive were obtained for Rheumatoid arthritis (HRA000155), and subsequently processed by cell ranger to obtain the count matrix.^67^ During the data selection stage, we specifically chose datasets that exhibited expression of canonical markers associated with myeloid cells, ensuring the availability of these markers for accurate downstream annotation. We also exclude datasets that posed challenges in extracting myeloid cell populations through the unsupervised clustering approach.

The single-cell data underwent processing for dimension reduction and unsupervised clustering using the workflow outlined in Scanpy.^55^ Quality control procedures were conducted for each dataset. Cells expressing fewer than 200 genes, less than 1000 total gene counts, and over 20% of mitochondrial genes were classified as low-quality cells and thus eliminated. To address doublets, cells expressing over 4000 genes and exceeding 30000 total counts were initially filtered out. Subsequently, Scrublet was employed to identify and remove predicted doublets as well as clusters enriched with doublets. Following quality control, we implemented the library-size correction method to standardize the raw counts, utilizing the scanpy.pp.normalize_total function within Scanpy. We conducted unsupervised clustering to retain myeloid cell clusters exhibiting elevated expression levels of CD68 and CST3 and removed those clusters who expressed both myeloid cell marker and lymphocytes marker (T cell: CD3E, B cell: MS4A1).

The bulk RNA datasets utilized to replicate the findings were sourced from the GEO database using GEOquery package^56^ with the following accessions^47^^,^^48^^,^^49^^,^^50^^,^^51^^,^^52^^,^^53^^,^^54^: Ankylosing Spondylitis (GSE25101), Multiple sclerosis (GSE203241), Psoriasis (GSE55201), Rheumatoid arthritis (GSE17755), Juvenile idiopathic arthritis (GSE17755), Sjogren Syndrome (GSE48378), Systemic lupus erythematosus (GSE49454), and Type 1 diabetes (GSE72377). The expression was normalized across the sample utilizing normalizeBetweenArrays() function implemented in R limma. An in-house SLE bulk RNA-seq data was used to validate the module 1 expression in patients at different disease stages.^53^

### Data integration and unsupervised clustering

We retained 11,957 genes that were consistently present across all samples for subsequent analysis. Initially, highly variable genes were chosen for downstream analysis using the scanpy.pp.highly_variable_genes function. Subsequently, the impact of total count per cell and the percentage of mitochondrial gene count were regressed out using the scanpy.pp.regress_out function. A principal component analysis (PCA) matrix was computed utilizing the scanpy.tl.pca function with the parameter svd_solver = 'arpack'. To remove the batch attribute to different samples and different datasets, we utilize scanpy.external.pp.harmony_integrate function setting sample id and the origin of the sample as the key. The resulting PCA corrected from harmony are utilized for the UMAP and clustering using scanpy.tl.neighbors, scanpy.tl.leiden and sc.tl.umap.^10^ The canonical marker is adapted from CellMarker database as reference for the first round of cell annotation.^68^

A second round of dimension reduction and clustering were conducted independently on the monocytes (classical and non-classical) as well as on the dendritic cells (classical and plasmacytoid). Another round of the dimensional reduction procedure was performed separately on the monocytes and dendritic cells. Marker genes for each cluster were identified using sc.tl.rank_genes_groups and visualized using R Nebulosa.^59^ To elucidate the functional significance of each cluster, gene ontology enrichment analysis was carried out upon the genes with adjust *p* value <0.05 and log2 fold change >0.3 using R clusterProfiler.^58^ The relationship between the cell subtypes were visualized using scanpy.pl.dendrogram.

We have built a portable reference based on our integrated dataset using Symphony.^60^ Harmony-corrected PCA was utilized to create the reference. This enables researchers to annotate their own myeloid cells using this atlas-level reference. The vignette and reference can be found here (https://github.com/FrankQYW/Myeloid_Autoimmune_Ref).

### Cell subgroup annotation

To measure the classical functions of myeloid cells, we acquired genes belongs to the HALLMARK_INTERFERON_GAMMA_RESPONSE, KEGG_ANTIGEN_PROCESSING_AND_PRESENTATION, HALLMARK_INTERFERON_ALPHA_RESPONSE, KEGG_FC_GAMMA_R_MEDIATED_PHAGOCYTOSIS from MSigDB database.^69^ We score cells with respect to specific signatures using scanpy.tl.score genes function.

The activity of regulons within each cell subtype is measured using SCENIC.^12^ Initially, the co-expression network was generated utilizing GRNBoost2, followed by the identification of regulons using RcisTarget. Subsequently, the activity of regulons for each cell was evaluated using AUCell.

To measure the cytokine responsiveness among the myeloid subsets, we first acquired pseudo bulk profile of each subset utilizing Python decoupler function.^65^ We then input the data into CytoSig to quantify the level cytokine responsiveness in each subset.^62^

#### Trajectory analysis

We utilized Monocle 3 with the UMAP embedding derived from the previous scanpy workflow.^13^ The “learn_graph” function was executed with the parameter “use_partition = FALSE” to construct a linear trajectory with default settings. To order the cells along the trajectory, we designated the ITGAM_cMo cluster as the root for inferring pseudotime based on the lowest expression of monocyte differentiation markers (MAFB, RHOC, CSF1R) with default parameters. Subsequently, we identified the top 1000 genes with the highest Moran’s I correlation score in relation to pseudotime using the graph_test function. Coexpressed modules were identified using the find_gene_modules function.

#### Functional analysis

In order to assess the varying regulation of coexpressed modules identified from previous analysis in monocytes across different autoimmunity conditions, we initially pinpointed the differentially expressed genes in monocytes by comparing samples from each disease condition to their respective controls using scanpy.tl.rank_genes_groups. Subsequently, gene set enrichment analysis (GSEA), applied through R clusterprofiler was conducted for each disease condition on the list of genes sorted by z-scores obtained from the differential expression analysis.^70^ Only diseases with available corresponding healthy control data in the primary study were included in this analysis.

Gene set variation analysis (GSVA), applied through the R GSVA package, is carried out on the normalized counts obtained from the bulk data.^61^ Module scores were calculated for each sample in each dataset. Wilcoxon rank sum test was employed to assess the variations in the GSVA scores of modules 1 between the patients and healthy controls.

### Protein-protein interaction network and drug repurposing

In order to build the protein-protein interaction network, the genes in co-expression module were imported into the Search Tool for the Retrieval of Interacting Genes (STRING).^63^ STRING infers interactions using evidence from high-throughput experiments, databases on Protein-Protein interactions (PPI), co-expression and co-occurrence of relevant genes. The minimum requirement score indicating the confidence of interaction was set to 0.4. The ChEMBL and DrugBank database, which are repositories of information on drugs and targets were used to identify the drug targets for the genes in the PPI network.^71^ We only selected the drugs that are in trials phases III and IV and originally target at least one type of autoimmune disease. The resulting network is visualized using Cytoscape.^66^

### Heritability analysis

GWAS summary statistics were obtained from the GWAS catalogue with the accession number provided in the supplementary material. Z-scores of feature genes for each cell subset were computed through differentially expression analysis using scanpy.tl.rank_genes_groups(). The top 10% of genes, ranked by the Z-score, underwent min-max normalization to generate a list of feature genes for each cell subset. CELLECT was utilized to prioritize the cell types enrich the heritability of a specific complex feature by combining the GWAS summary statistics with the list of feature genes.^64^ Briefly, CELLECT quantifies the relationship between common polygenic GWAS signals (heritability) and the expression specificity of genes in cell types using stratified linkage disequilibrium score regression (S-LDSC). Cell subtype with FDR <0.1 were retained.

### Quantification and statistical analysis

All statistical analysis is performed in R and Python. The comparison of the GSVA score between the conditions is performed by the wilcoxon rank sum test. All other statistical analyses are detailed in the respective sections of the article.

## Supplemental information

Document S1. Figures S1–S6 and Tables S1 and S2

Table S3. Essential functional modules in monocytes identified in this study, related to Figure 5

Table S4. GWAS studies used in the heritability analysis, related to Figure 6

Table S5. Cell origin of the dataset, related to Figure 1
