# Supplementary material for: Single-cell transcriptional landscape of peripheral myeloid cells in autoimmune diseases
Source: iScience. 2025 Jul 1;28(8):113026. doi: 10.1016/j.isci.2025.113026 (PMC12283746; doi:10.1016/j.isci.2025.113026)
Supplement: Document S1. Figures S1–S6 and Tables S1 and S2 [file mmc1.pdf]

iScience, Volume *28*

Supplemental information

Single-cell transcriptional landscape of peripheral myeloid cells in autoimmune diseases

Frank Qingyun Wang, Xiao Dang, Caicai Zhang, Huidong Su, Yao Lei, Xinxin Chen, Jing Yang, and Wanling Yang

A

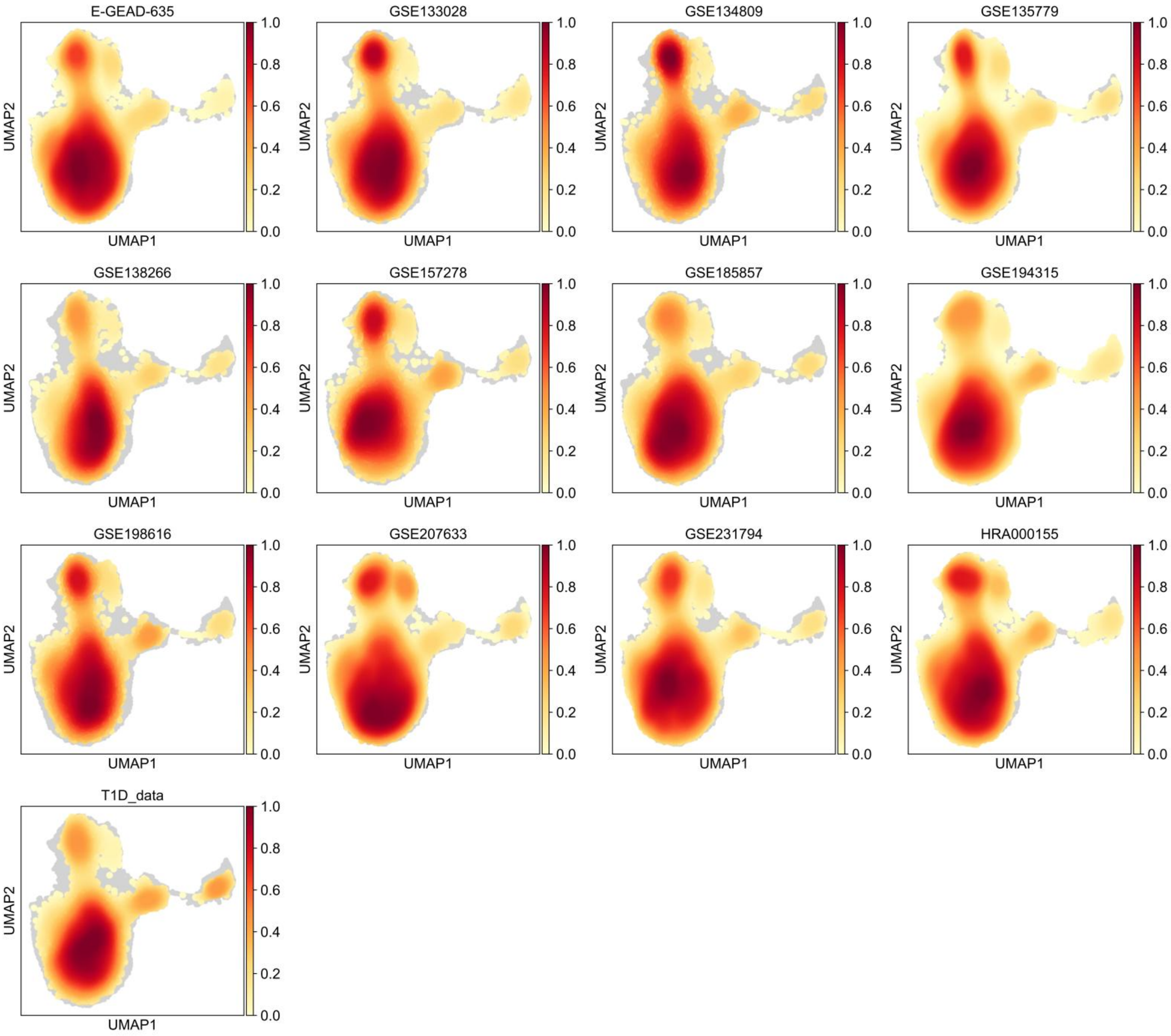

Figure S1. Density plot showcasing distribution of cells from different resources on UMAP embedding, related to Figure 1.

(A) Density plot shows that the dataset origin of cells are evenly distributed in the UMAP.

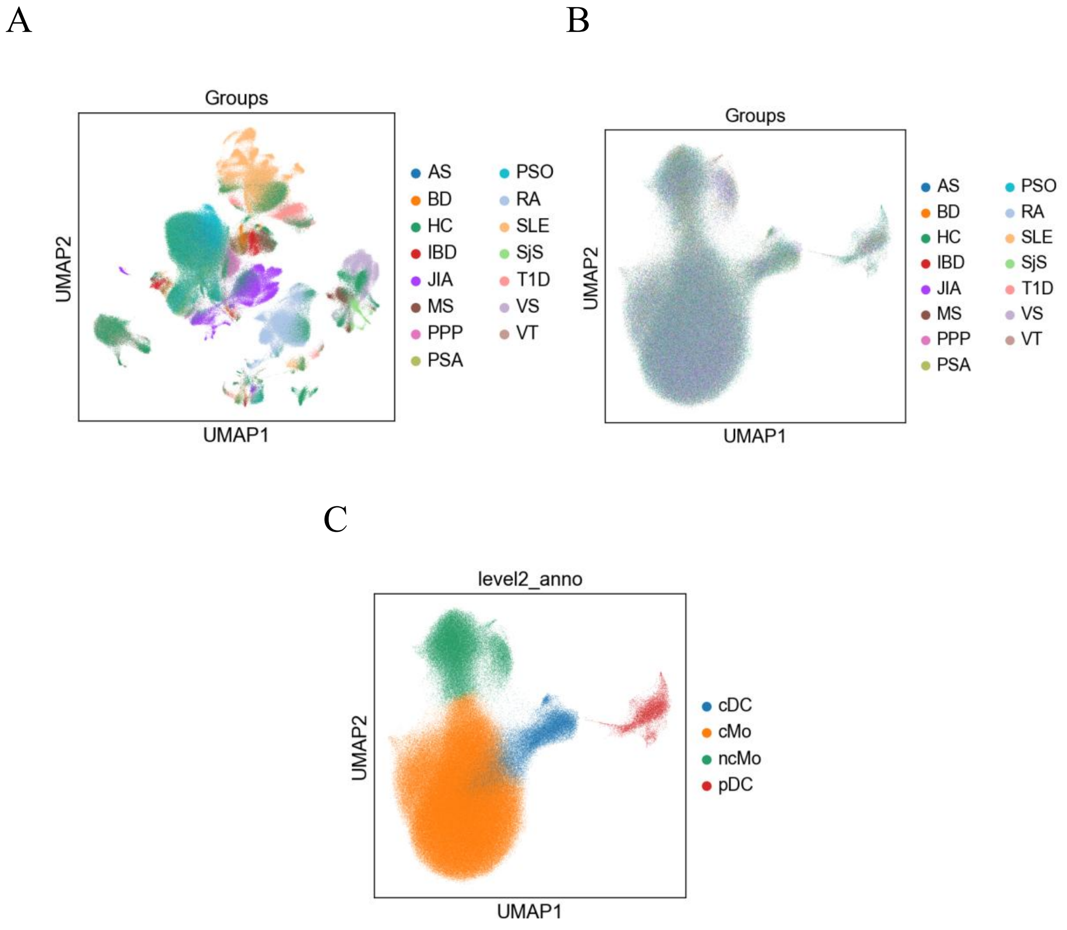

Figure S2. Harmony correct the batch effect across different studies, related to Figure 1.

(A). UMAP of myeloid cells without correcting the batch effect using harmony grouped by the disease condition. (B-C). UMAP of myeloid cells with batch effect corrected grouped by the disease and cell types.

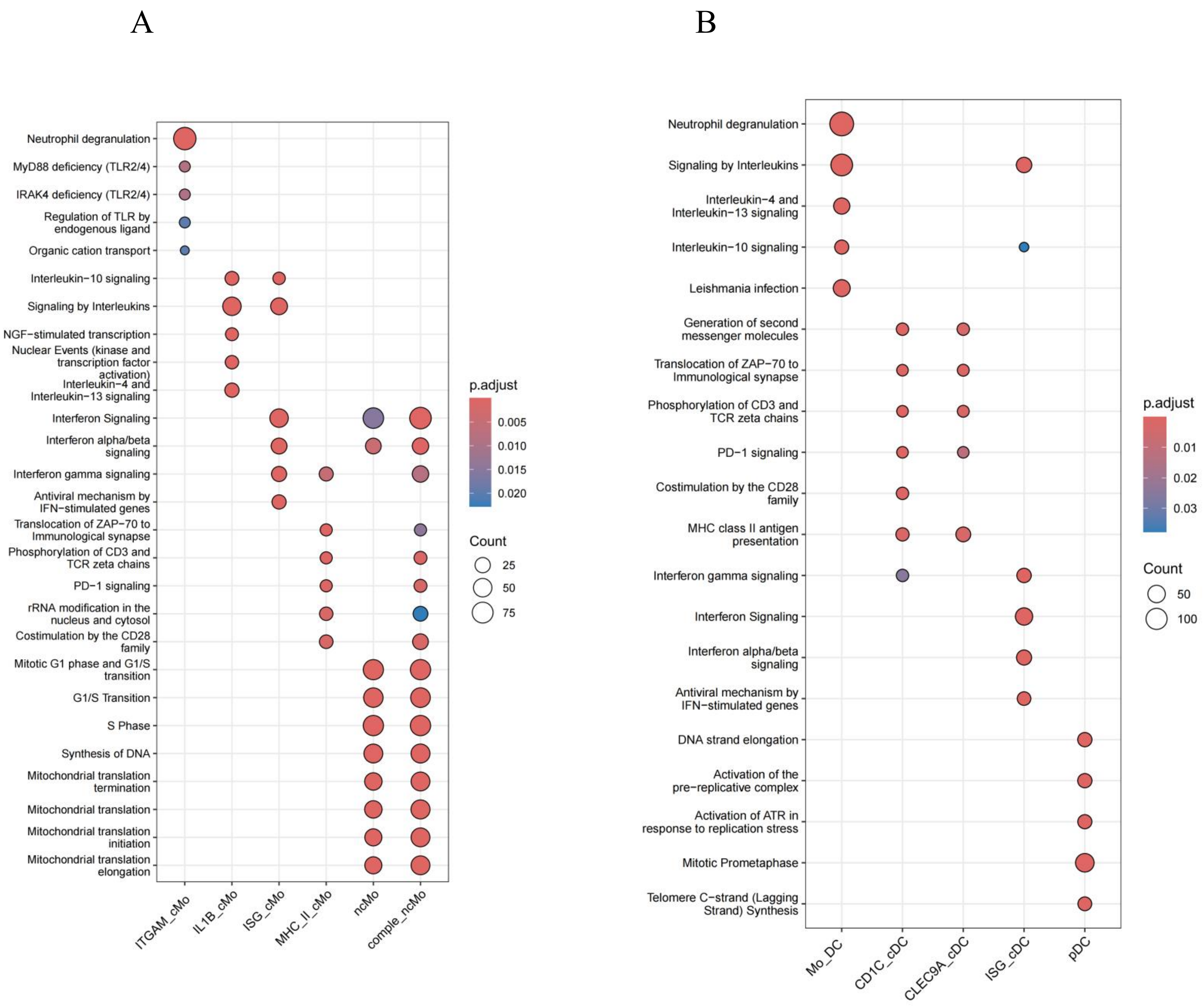

Figure S3. Functional enrichment analysis of monocyte and dendritic cell subsets, related to Figure 2 and Figure 3.

(A) Dotplot showing the enrichment results of monocyte subsets using Reactome terms. (B) Dotplot showing the enrichment results of dendritic cell subsets using Reactome terms.

A

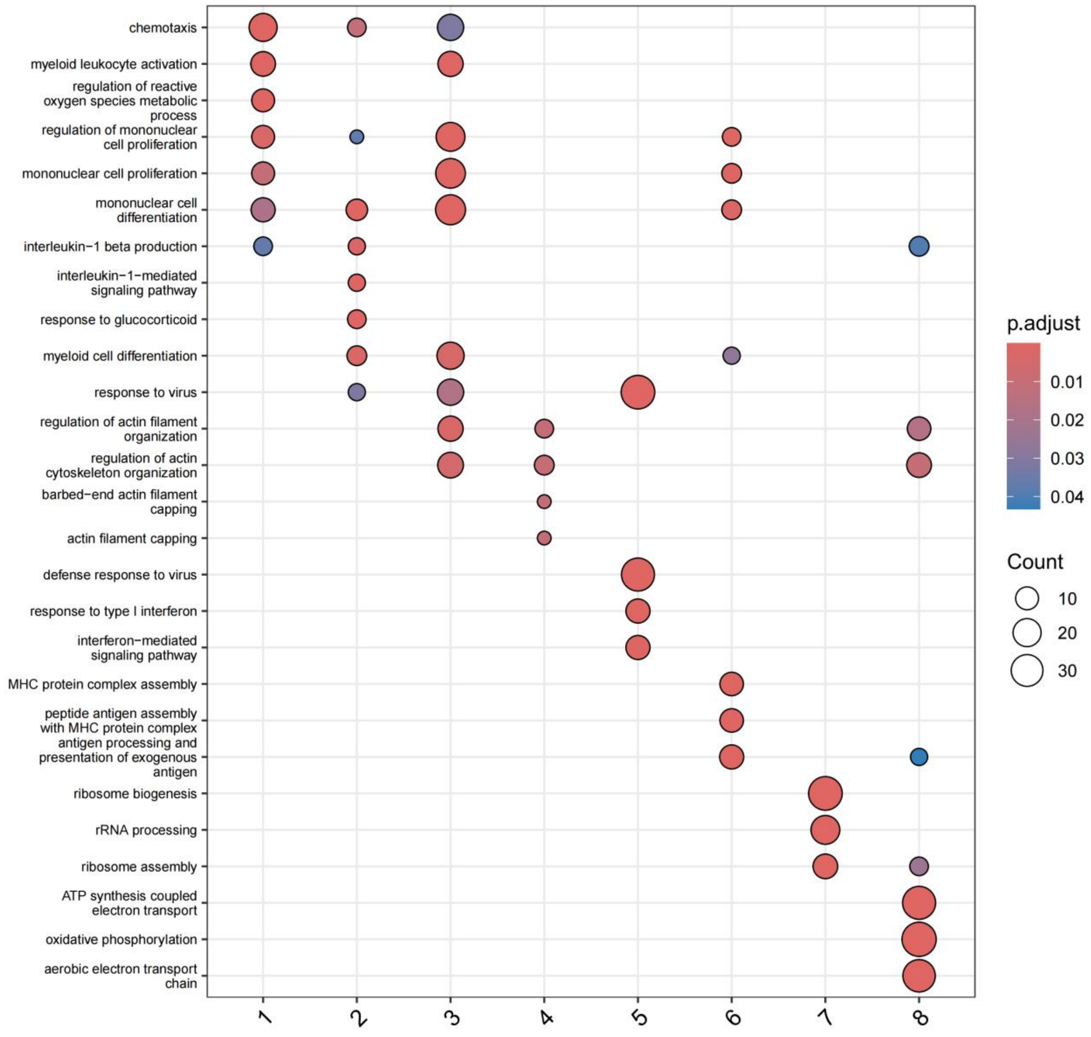

Figure S4. Functional enrichment analysis of 8 co-expression modules, related to Figure 5.

Dotplot showing the p.adjust value (color) and gene counts (size) of each enriched terms in each module.

A

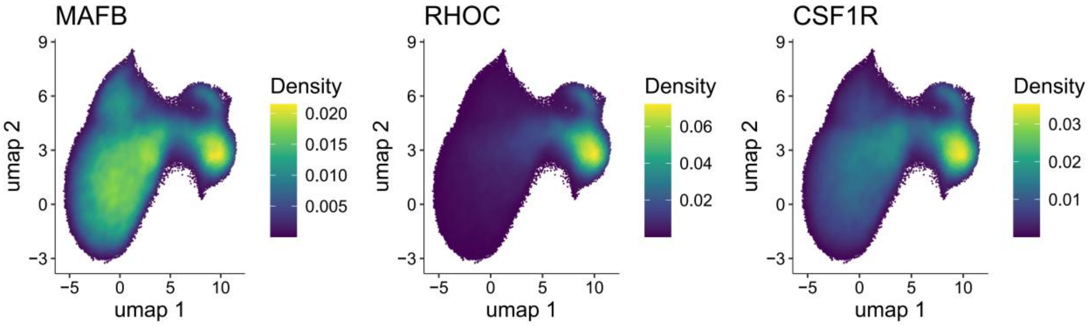

B

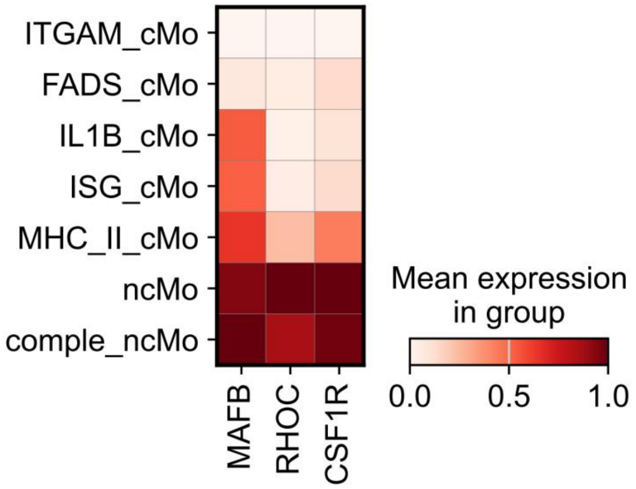

Figure S5. Expression of known monocyte differentiation markers, related to Figure 5.

(A) UMAP plot showing the expression of monocyte differential markers.

(B) Heatmap plot showing the expression of monocyte differential markers in monocyte subsets. Cells with lower expression represent the state with higher differential potential.

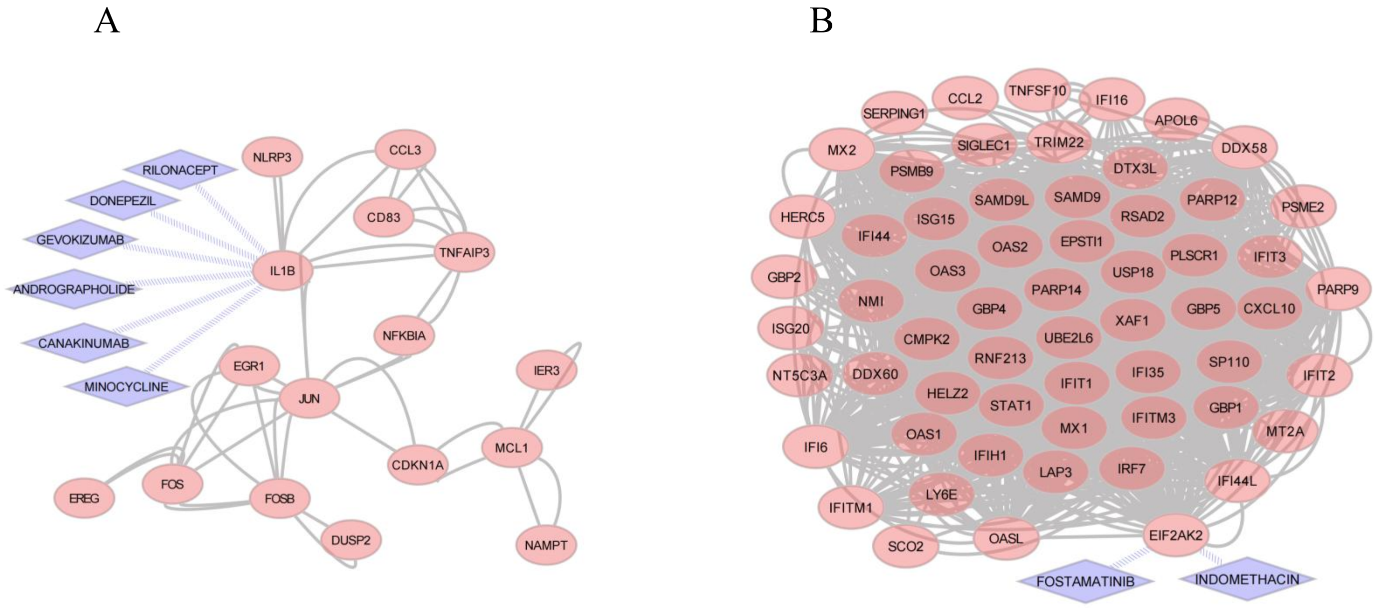

Figure S6. Protein-protein interaction network of module 2 and module 5, related to Figure 6.

(A-B) PPI network showing the genes connected and their potential drug targets in module 2 (left) and module 5 (right).

Accession	Database	Disease_Type
GSE231794	Gene Expression Omnibus	Vitiligo
GSE185857	Gene Expression Omnibus	Palmoplantar Pustulosis
GSE138266	Gene Expression Omnibus	Multiple Sclerosis
GSE133028	Gene Expression Omnibus	Multiple Sclerosis
GSE194315	Gene Expression Omnibus	Psoriasis + Psoriatic arthrtis + Ankylosing spondylitis
HRA000155	Genome Sequence Archive	Rheumatoid Arthrtitis
E-GEAD-635	Genomic Expression Archive	Vasculitis
GSE198616	Gene Expression Omnibus	Behcet Disease
GSE207633	Gene Expression Omnibus	Juvenile idiopathic arthritis
GSE135779	Gene Expression Omnibus	Systemic lupus erthymatosus
GSE157278	Gene Expression Omnibus	Sjogren syndrome
syn53641849	Synapse	Type 1 diabetes
GSE134809	Gene Expression Omnibus	Crohn’s disease

Table S1. Single cell RNA-seq dataset used in this study, related to Figure 1.

Cell type	Marker
Myeloid cells	CST3,CD68
Classical Monocyte	CD14
Non-classical Monocytes	FCGR3A
classical dendritic cell	CD1C
plasmacytoid dendriti cell	LILRA4

Table S2, Canonical markers used in this analysis, related to Figure 1.
